# Influence of Different Envelope Maskers on Signal Recognition and Neuronal Representation in the Auditory System of a Grasshopper

**DOI:** 10.1371/journal.pone.0034384

**Published:** 2012-03-30

**Authors:** Daniela Neuhofer, Bernhard Ronacher

**Affiliations:** 1 Department of Biology, Humboldt-Universität zu Berlin, Berlin, Germany; 2 Bernstein Center for Computational Neuroscience Berlin, Berlin, Germany; Imperial College London, United Kingdom

## Abstract

**Background:**

Animals that communicate by sound face the problem that the signals arriving at the receiver often are degraded and masked by noise. Frequency filters in the receiver's auditory system may improve the signal-to-noise ratio (SNR) by excluding parts of the spectrum which are not occupied by the species-specific signals. This solution, however, is hardly amenable to species that produce broad band signals or have ears with broad frequency tuning. In mammals auditory filters exist that work in the temporal domain of amplitude modulations (AM). Do insects also use this type of filtering?

**Principal Findings:**

Combining behavioural and neurophysiological experiments we investigated whether AM filters may improve the recognition of masked communication signals in grasshoppers. The AM pattern of the sound, its envelope, is crucial for signal recognition in these animals. We degraded the species-specific song by adding random fluctuations to its envelope. Six noise bands were used that differed in their overlap with the spectral content of the song envelope. If AM filters contribute to reduced masking, signal recognition should depend on the degree of overlap between the song envelope spectrum and the noise spectra. Contrary to this prediction, the resistance against signal degradation was the same for five of six masker bands. Most remarkably, the band with the strongest frequency overlap to the natural song envelope (0–100 Hz) impaired acceptance of degraded signals the least. To assess the noise filter capacities of single auditory neurons, the changes of spike trains as a function of the masking level were assessed. Increasing levels of signal degradation in different frequency bands led to similar changes in the spike trains in most neurones.

**Conclusions:**

There is no indication that auditory neurones of grasshoppers are specialized to improve the SNR with respect to the pattern of amplitude modulations.

## Introduction

During evolution a variety of auditory systems evolved, whose major task is the detection and classification of behaviourally relevant sounds. Conclusions about what is happening in the acoustic environment can only be inferred from sequences of action potentials, which encode important features of the acoustic signal. However, on their way from the sender to a receiver, signals are usually modified by extrinsic noise, which degrades the information available for the receiving nervous system. Extrinsic noise has many different sources [1], [2], [3], [4] and can affect signal detection as well as signal recognition [5], [6], [7]. Signal recognition is impeded by modifications in the temporal structure and in the spectral content of a signal. Acoustic measurements have shown that atmospheric turbulence or temperature gradients give rise to unpredictable amplitude fluctuations of signals, mostly in the frequency range below 50 Hz [1], [4], [8]. Since natural signals often include amplitude modulations within this range, this type of degradation could have a major impact on signal recognition [9]. Another problem for signal detection and recognition is auditory masking due to biotic background noise from other singing individuals, whether conspecifics or heterospecifics [10]. This structured acoustic background by nature has similar modulation frequencies as the signal to be detected. The receiver therefore has to be capable of extracting behaviourally relevant signals from the interfering noise [10], [11], [12], [13].

These problems must be particularly severe for organisms that use broadband communication signals and that have ears with poor spectral resolution, because they cannot rely on peripheral frequency filtering to reduce extrinsic noise. As a consequence, these organisms have to rely on other strategies to enable signal recognition in noisy habitats. If the noise is not correlated along the carrier frequency spectrum, a sampling over a wide range of frequencies could help to sustain signal recognition in noise [5], [14]. We hypothesize that also a filtering mechanism in the time domain could sustain signal recognition in noisy habitats.

Compared to vertebrates, the frequency resolution in acridid grasshoppers is poor [15]. However, the auditory receptors show a high temporal precision and are able to reliably reflect the temporal details of the stimulus envelope [16]. Indeed, the recognition of species and sex is primarily based on temporal cues present in the amplitude modulations of the song envelope which, for example give rise to a characteristic syllable pause structure of the acoustic signal [17], [18]. Studies using sinusoidal amplitude modulated (SAM) stimuli have shown that auditory receptors and several local interneurons of *Locusta migratoria* exhibit all-pass behaviour in their spike rates. In contrast, ascending neurons, which transmit the auditory information to the decision centres located in the brain, show low-pass or band-stop characteristics [19], [20], [21]. The filter ranges of these neurons correspond well to the major amplitude modulations found in grasshopper songs, which encompass frequencies between 10 and 100 Hz [22]. So far it has been argued that, above all, the neurones' selectivity for distinct amplitude modulations is important for pattern recognition; but cells responding selectively to certain modulation frequencies could also have the potential to decrease interfering amplitude modulations caused by extrinsic noise. Whether a filter mechanism in the domain of modulation frequencies would really be able to improve the signal-to-noise ratio by reducing the noise components is, however, not clear.

This study aimed at testing whether envelope maskers with their energy concentrated within the species-specific frequency range could have a stronger impact on signal degradation than envelope maskers outside this frequency range. Using the same stimuli both in behavioural tests and neurophysiological recordings, we investigated the effects of interfering amplitude modulations within different frequency ranges on signal recognition as well as on the neuronal representation of these communication signals.

## Materials and Methods

### Ethics statement

The experiments reported in this paper comply with the current laws for animal protection in Germany: no specific permits are required for studies on insects.

### Animals

Behavioral experiments were performed on adult males of *Chorthippus biguttulus.* Intracellular recordings were performed on adult individuals of *C. biguttulus* and *Locusta migratoria*. Since two earlier studies [23], [24] have revealed no differences between neurons of the early auditory pathway, locusts were used to complement the electrophysiological data of *C. biguttulus*. Males and females were used for electrophysiology as no sex-specific differences were found for auditory neurons in the metathoracic ganglion (own data and Stumpner – personal communication). The locusts were obtained from a commercial supplier, *C. biguttulus* were caught in the field or F1 reared from our own breeding stock.

### Acoustic Stimuli

**Figure 1:**
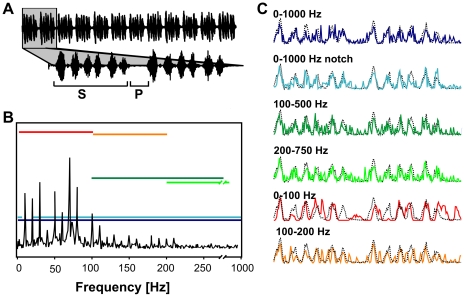
Model songs used for behavioural and neurophysiological tests. A) The upper panel shows the oscillogram of the original female song which contained 12 similar syllables (S) of 80 ms length separated by pauses (P) of around 20 ms. The lower panel shows the enlargement of two syllables. Each syllable consisted of 6 sound pulses B) Amplitude spectrum of the envelope of the original female song. The frequency ranges of the different bands of envelope degradation, which were used both in behavioural experiments and intracellular recordings are indicated with coloured, horizontal bars. C) The envelopes of two song syllables before (dotted black line) and after adding envelope noise at 0 dB NSR (colored line).

For behavioural and neurophysiological experiments six envelope maskers containing different modulation frequency ranges were used (0–1000 Hz, 0–1000 Hz with notch between 5 and 20 Hz, i.e. leaving the range of the fundamental modulation frequency of the female song unharmed, 0–100 Hz, 100–200 Hz, 100–500 Hz, 200–750 Hz; see Figure 1B+C). These maskers were chosen for the following reasons: The most interesting AM frequency border is located at ∼100 Hz, since the AM frequencies of the species-specific female song encompass mainly the range between 10 and 100 Hz. Thus, our maskers aimed at covering or sparing the 10–100 Hz range. However there were additional indications that higher frequencies may have a negative impact on signal attractiveness [17]. With the 100–500 Hz and 200–750 Hz bands we wanted to test for an influence of relatively high frequencies, with maskers that differed in the 100–200 Hz range. The low frequency range was then further explored by the comparison of the 0–100 Hz and 100–200 Hz maskers. In addition, we asked whether the fundamental frequency of the song (∼10 Hz) was particularly susceptible and for this reason we compared the broad band maskers (0–1000 Hz) and (0–1000 Hz notch). It was only after our experiments were begun, that Schmidt et al. [18] showed for *C. biguttulus* females that the fundamental frequency can be omitted, without affecting the attractiveness of song models.

The signal generation is described in detail elsewhere [25]. The data for the 0–1000 Hz noise band were reported also in [25], in a different context. The envelope noise was identical across subsequent trials for a given degradation level (“frozen noise”), whereas for each successive level a new stochastic degradation was generated. Signal degradation was performed in 3 dB steps relative to the variance of the original envelope. The noise-to-signal ratio (NSR) is given in decibels by NSR = 10 log (noise variance)/(original song variance). As a consequence, a degradation level of 0 dB refers to a noise-to-signal ratio of one. The resulting envelopes were filled with the typical carrier frequency spectrum of female songs [25], [26]. Since the total noise energy per degradation level was equal for each envelope masker, the different bandwidths lead to different noise amplitudes at the frequency ranges involved. This is diagrammed in Figure 1B.

### Behavioral tests

The playback experiments used to quantify song recognition in *C. biguttulus* are described in detail elsewhere [25], [27]. Basically, a *C. biguttulus* male was stimulated with female model songs in a pseudorandom order via a laterally situated speaker and the phonotactic response was registered. Each stimulus was repeated at least 10 times. The response probability for each stimulus class was calculated as the percentage of phonotactic responses relative to the total number of stimulus presentations. A behavioural critical degradation level (bCDL) was determined as the degradation level at which the behavioural response dropped to below 50%. The distributions of critical degradation levels for different envelope bands were compared using the Kruskal-Wallis H-Test. Stimulus attractiveness was compared applying Fisher's exact test with Yates and Bonferroni-Holm correction.

### Neurophysiology-intracellular recordings

The preparation and the intracellular recordings were conventional and are described in detail elsewhere [28]. During the experiments the preparation was kept at a constant temperature (30±2°C). All experiments were performed in a Faraday cage lined with foam prisms to reduce echoes. Intracellular recordings were obtained from auditory interneurons within the metathoracic ganglion using thin-walled glass capillaries, the tips of which were filled with 0.5 M LiCl and 3–5% Lucifer yellow (Sigma–Aldrich, Taufkirchen, Germany). Neural responses were amplified (Bramp-01; npi electronic, Tamm, Germany) and recorded by a data-acquisition board (PCI-MIO-16E-1; National Instruments, Munich, Germany) with a sampling rate of 20 kHz. After completion of the stimulation protocol the dye was iontophoretically injected into the recorded neuron. The thoracic ganglia were removed, fixed in 4% paraformaldehyde, dehydrated, and cleared in methylsalicylate. The stained cells were identified according to their characteristic morphology [29], [30].

### Neurophysiology - Stimulation

Acoustic stimuli were stored digitally and delivered by custom-made software (LabVIEW, National Instruments). Following a 100-kHz D/A-conversion, the stimulus was routed through a computer controlled attenuator (PA5; Tucker-Davis Technologies, Gainesville, FL) and an audio amplifier (Mercury 2000; Jensen, Pulheim, Germany). Acoustic stimuli were broadcast by speakers (D-28/2, Dynaudio, Skanderborg, Denmark) situated a distance of 30 cm from the preparation. Depending on the directionality of the recorded neuron, the stimulus was given unilaterally from the right or from the left side. Sound intensity was calibrated with a ½-inch microphone (type 4133, Brüel & Kjær) and a measuring amplifier (type 2209, Brüel & Kjær), positioned at the site of the preparation. The sound intensity was set to 60 dB SPL and each stimulus was repeated 10 times.

### Determining spike train similarity

We computed a metric distance between pairs of spike trains according to van Rossum [31]. Spike times were extracted from the digitized recordings, and each spike was convolved with an α-function filter [32]. The width of the filter function was set by the time constant τ to 5 ms (compare [25]). The pairwise differences of the convolved spike train traces were computed. The spike train distance resulted from the root-mean-squared integral of the pairwise differences. To allow a comparison between different cell types that produce different spike rates, spike train distances were normalized by the mean spike count in response to the original song. To quantify the impact of external envelope degradation on neuronal representation we computed the slopes from the linear regression of the mean distance values for each degradation level (see Results section for details). As test for statistical significance of differences in distance slopes we used the the Wilcoxon signed rank test. In case of multiple comparisons a Bonferroni correction was applied.

## Results

By a combination of behavioural and neurophysiological experiments, this study aimed to investigate the filter capacities of the grasshopper auditory system for envelope noise. For this purpose a species-specific female song (Figure 1A) was used, whose envelope was degraded by adding random amplitude modulations. The main amplitude modulations of female songs cover a narrow frequency range between 10 and 100 Hz (Figure 1B). If the auditory system is capable of filtering out interfering amplitude modulations, envelope maskers with their energy concentrated in this relevant frequency range should degrade the song signals more efficiently than maskers outside this frequency range. We used six envelope maskers containing different modulation frequency ranges to test this hypothesis. For a more detailed illustration of this approach, Figure S1 illustrates the fast Fourier transform (i.e. the frequency composition of the envelope) of the signal used, degraded with four of our envelope maskers.

### Influence of signal degradation on signal recognition – playback experiments

The effect of external envelope degradation on signal recognition was quantified in males of *Chorthippus biguttulus*, by taking advantage of their phonotactic response. The first step of phonotaxis - a conspicuous turning movement towards the signal of a species-specific female - is a reliable indicator of signal recognition [25], [26], [33].

**Figure 2:**
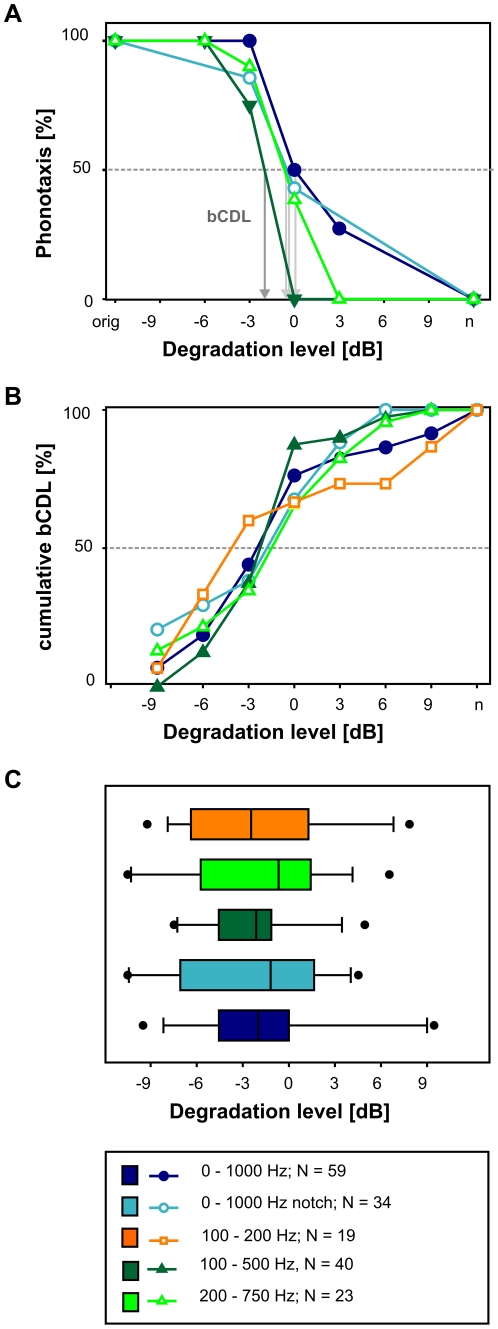
The influence of different envelope maskers on signal recognition. A) Turning response of one male tested with four different degradation bands. The abscissa shows the degradation levels in dB, ‘orig’ indicates the original song; ‘n’ indicates pure noise. The ordinate shows the percentage of turning responses. For each phonotaxis curve a behavioural critical degradation level (bCDL) was interpolated at the intersection of each phonotaxis-curve with the 50% response level. B) Cumulative percentage of bCDLs for five different bands of envelope degradation. The ordinate shows the cumulative bCDLs [in %] as a function of signal degradation (abscissa). C) The distribution of bCDLs for five of six frequency bands tested. Boxes cover the interquartile ranges, whiskers the range from the 10th to the 90th percentile. Points indicate the outliers The medians bCDLs of these bands were between-3 dB and 0 dB. 0–1000 Hz: median = −2 dB, N = 59; 0–1000 Hz notch: median = −1.2 dB N = 34, 100–500 Hz: median = −2.1 dB, N = 40, 200–750 Hz: median = −0.7 dB, N = 23; 100–200 Hz: median = −2.5 dB, N = 19.

Figure 2A exemplifies the effect of different bands of envelope noise on signal recognition by the representative response behaviour of a *Chorthippus biguttulus* male. Every presentation of the uncorrupted female song elicited a turning response, yielding a response rate of 100%. Between −3 dB and 3 dB the response probability dropped sharply. Remarkably, there was little difference between the degradation bands tested. A behavioural critical degradation level (bCDL) was determined as the degradation level at which the behavioural response dropped to 50% (see arrows in Figure 2A). For this individual, the bCDL was around 0 dB for 0–1000 Hz, 0–1000 Hz with notch and 200–750 Hz. The bCDL for the 100–500 Hz band was shifted to a slightly lower degradation level (−2 dB). A cumulative plot of the critical degradation levels of many individuals indicates that for all frequency bands signal recognition became severely impaired at degradation levels between −6 and 0 dB (steepest slopes in Figure 2B, see legend for sample sizes). A Kruskal-Wallis test revealed no significant differences between the bCDL distributions (Figure 2C, p = 0.95). These results demonstrate that different envelope maskers had similar effects on signal recognition, regardless of their degree of frequency overlap with the amplitude spectrum of the original signal.

**Figure 3:**
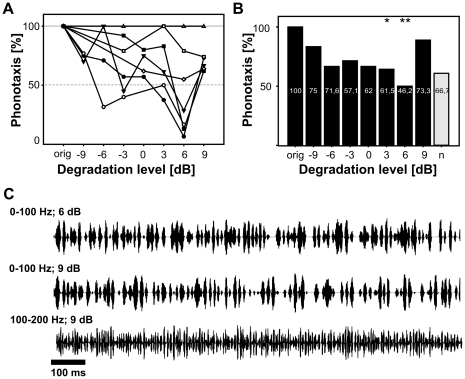
The influence of the 0–100 Hz envelope masker on signal recognition. A) Phonotaxis response (ordinate) of seven males as a function of degradation level (abscissa) B) Attractiveness for each degradation level (abscissa) measured as the proportion of animals that responded in more than 50% of the trials (ordinate). Numbers within the bars indicate the median response probabilities. Altogether 20 males were tested, the sample sizes for the individual degradation levels were: −9 dB: N = 12, −6 dB: N = 9, −3 dB: N = 14, 0 dB: N = 12, 3 dB: N = 14, 6 dB: N = 12; 9 dB: N = 18. The pure noise (n) was tested on a different set of animals; N = 23. Asterisks mark significant differences between the original song and degraded songs (Fisher's exact test with Yates and Bonferroni-Holm correction). C) The upper and the middle panel show the oscillograms of the original female song degraded with 0–100 Hz at 6 and 9 dB respectively. The lower panel illustrates the degradation with 100–200 Hz at 9 dB degradation level.

However, there was one exception from this rule: for the signal degradation with amplitude modulations between 0–100 Hz it was not possible to compute a critical degradation level (bCDL) because the animals showed no consistent reduction of turning responses to this envelope masker. The response behaviour is exemplified for seven males in Figure 3A. In contrast to the results of all other envelope maskers, the percentages of phonotaxis response did not decrease progressively with increasing levels of signal degradation (compare with Figure 2A). There were several animals whose response probability never fell below the 50% criterion. Others, for which the 50% threshold was met at earlier degradation levels, showed a subsequent rise above this threshold at higher degradation levels. For 17 of the 20 animals tested, no clear bCDL could be determined. Figure 3B illustrates the stimulus attractiveness at successive degradation levels, which is defined as the proportion of males that responded to a distinct degradation level in more than 50% of all presentations (the definition of stimulus attractiveness follows [18]). The overall attractiveness was above 50% for all degradation levels but one (6 dB). This result was surprising, since this envelope masker had the strongest overlap with the main amplitude modulation frequencies of female songs (compare Figure 1B). One would expect that a noise band with its energy concentrated in the signal's frequency range would have a higher impact on signal recognition than envelope maskers outside this frequency range. The peculiar behaviour can probably be attributed to the effect that the degradation with amplitude modulations between 0–100 Hz by chance yielded pattern sequences that resembled the natural signals, and, therefore, were categorized as attractive (see discussion). Indeed, the oscillograms of songs degraded with 0–100 Hz show episodes of pulse-like structures which resemble the syllable structure of the original song (compare Figures 1 and 3C). Therefore, this hypothesis was further examined in an additional experiment, by presenting stimuli consisting of pure random amplitude modulations between 0 and 75 Hz. These stimuli also elicited rigorous phonotactic responses in 14 out of 23 tested males (see column **n** in Figure 3B).

In summary, the behavioural tests with degraded signals revealed that five of the six tested envelope maskers did affect signal recognition to a very similar amount. The only exception, a 0–100 Hz noise band, did not provide an efficient masker.

### Influence of signal degradation on neuronal signal representation – intracellular recordings

As a next step, we investigated whether a neuronal correlate can be found for the homogeneous effects of different envelope maskers above 100 Hz. Auditory neurons of the metathoracic pathway (i.e. local- and ascending interneurons, LN and AN) were investigated with respect to their potential filter capacities for envelope noise. We hypothesized that neurons with a higher filter capacity for envelope noise should be able to reduce the noise component of a degraded signal, resulting in less degraded spike trains in comparison to spike trains in response to a not degraded signal. For acoustic stimulation, the same stimuli as in the behavioural experiments were used (see Figure 1).

**Figure 4:**
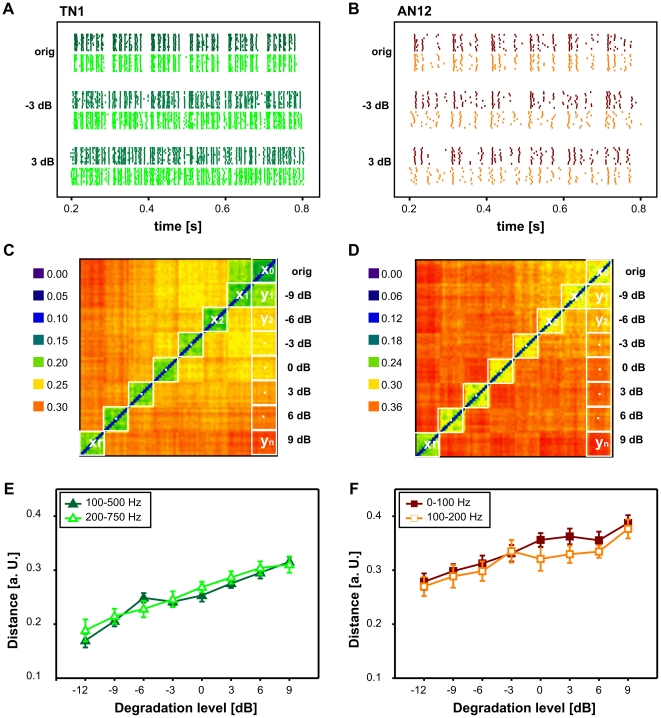
Quantification of the spike train degradation due to different envelope maskers. A) Spike train patterns from the local interneuron TN1 in response to the original song, −3 dB and 3 dB signal degradation. The degradation with 100–500 Hz is shown in dark green (*upper traces*). The degradation with 200–750 Hz is shown in light green (*lower traces*). B) Spike train patterns from the ascending interneuron AN12. Again, the responses to different envelope maskers are colour coded (0–100 Hz in dark red, 100–200 Hz in orange). C) Distance matrix of the TN1 for 100–500 Hz signal degradation. Metric distances are colour coded from blue to red (low to high distance). Square blocks along the diagonal contain the intrinsic distance values for each degradation level (e.g. x0: distances between spike trains in response to the original song). Squares along the right column indicate extrinsic distance values (e.g. y1 for distances between spike trains in response to the original song and the first degradation level). (Distances were normalized by the respective mean spike counts, see Material and Methods.) D) Distance matrix of the AN12 in response to 0–100 Hz signal degradation. E) Average values (± standard deviation) of spike train distances between spike trains in response to the original song and progressively degraded songs for the TN1. The abscissa shows the degradation level in dB, the ordinate the spike train distance in arbitrary units. Again, the distance curves for two different envelope maskers are shown in the same colours as in A (see inset). F) Distance curves for the AN12.

Figure 4A shows the spike raster plots of a local interneuron (TN1) tested with two maskers (100–500 Hz and 200–750 Hz). The spike trains in response to the original song and two degradation levels (−3 dB, 3 dB) are shown; each identical stimulus was presented ten times. Once random amplitude modulations were added to the original signal, the rhythmic pattern of the spike trains, which mirrors the fine structure of the envelope, got increasingly distorted. To quantify these distortions, pairwise distances between all spike trains were computed according to a spike train metric [31]. The resulting distances between every spike train and all other spike trains in the TN1 response are summarized for the 100–500 Hz band in a colour coded distance matrix in Figure 4C. Squares along the diagonal (x_0_, …, x_n_) represent spike train distances that resulted from repeated presentations of the same stimulus, i.e. distances due to trial-to-trial variability. The rightmost column of the distance matrix which is accentuated by eight vertically aligned squares represents distances between spike trains in response to the not degraded stimulus and spike trains in response to various degradation levels y_i_, that is, this column represents the impact the signal degradation had on the neuron's spike pattern.

To compare the influence of different envelope maskers on signal representation, average distances between spike trains in response to the original song and progressively corrupted songs were computed. The resulting distance curves for 100–500 Hz and 200–750 Hz envelope degradation are shown in Figure 4E. Both curves start from a similar, non zero distance value in response to the original song and exhibit a similar increase for successive levels of envelope degradation, revealing a comparable influence of the two envelope maskers on the neuronal representation. Similar results were found for an ascending neuron (AN12) which was tested with 0–100 Hz and 100–200 Hz envelope degradation. This cell responded with precise spikes at the syllable onset whereas the spikes in response to the further modulations within the syllables were more variable. For this cell as well, raising the noise level resulted in an increased distortion of spike responses. Again, the increase in spike train distances with increasing levels of signal degradation was similar for both maskers. Only for intermediate degradation levels (0 and 3 dB), there was a small difference between the two envelope maskers tested (Figure 4F).

**Figure 5:**
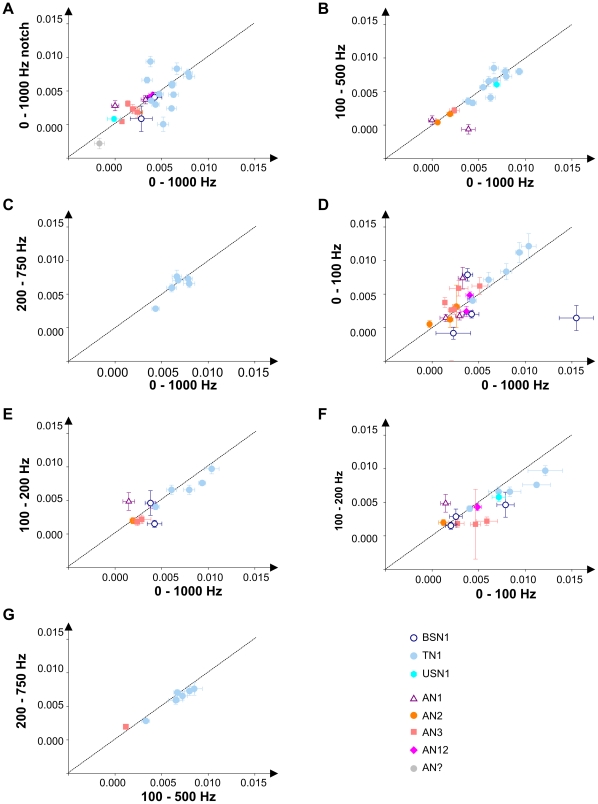
Comparison of the influence of different envelope maskers on neuronal representation. The graphs show the slopes of the distance curves (as in Fig. 4 E,F) of single cells, which were tested with different envelope maskers. Error bars indicate the mean standard error (s.e.m.) of the linear regression, each point in a graph represents a single neuron. The different cell types are indicated by different symbols (see inset). A) 0–1000 Hz vs. 0–1000 Hz notch, LN: 18, AN: 9; B) 0–1000 Hz vs. 100–500 Hz, LN: 12, AN: 5; C) 0–1000 Hz vs. 200–750 Hz, LN: 7; D) 0–1000 Hz vs. 0–100 Hz, LN: 9, AN: 13; E) 0–1000 Hz vs. 100–200 Hz, LN: 7, AN: 5; F) 0–100 Hz vs. 100–200 Hz, LN: 9, AN: 6; G) 100–500 Hz vs. 200–750 Hz, LN: 7, AN: 1.

In general, the investigated cells exhibited a linear increase of spike train distances with increasing levels of envelope degradation Therefore the slopes of the distance curves, as in Fig. 4E,F, could serve as a measure of the impact an envelope degradation had on the neuronal representation of the original female song (see Material and Methods, and [25]). By comparing the slopes of various distance curves we explored whether the different envelope maskers differed in their influence on neuronal signal representation. The graphs in Figure 5 summarize the pair-wise comparisons between these slopes for two envelope maskers each. Specimens of the different cell types are marked with different symbols (see inset). The local interneurones exhibited on average higher slope values than the ascending interneurones. Within a computation level there was no consistent difference between the different cell types investigated. For most comparisons, there were only minor, not significant deviations from the diagonal, indicating a similar impact of different envelope maskers. Table 1 summarizes the p values of the Wilcoxon signed rank test to compare the slopes of the distance curves in response to different envelope maskers. For local interneurons no comparison revealed significant differences between the slopes (taking a Bonferroni correction for multiple testing into account, the p level was set at 0.007). However, the 0–100 vs. 100–200 and the 100–500 vs. 200–750 Hz comparisons just missed this significance level. In both cases, the envelope maskers with higher modulation frequencies yielded lower slopes of the distance curves. Remarkably, even the ascending neurons did not show significant differences between different envelope maskers. This result was unexpected since the ascending neurons were the most likely candidates able to filter out high frequency envelope degradations, in view of the filter properties of their modulation transfer functions [19], [20], [21], see discussion.

**Table 1:** Results of the Wilcoxon signed rank test to pairwise compare the influence of different envelope maskers on neuronal representation.

Envelope maskers	LN	AN
**0–1000 Hz/0–1000 Hz notch**	**0.17** (N = 17; 2*BSN1, 13*TN1, 2*USN1)	**0.314** (N = 9; 2*AN1, 1*AN2, 4*AN3, 2*AN12)
**0–1000 Hz/100–500 Hz**	**0.347** (N = 12; 11*TN1, 1*UGN1)	**0.345** (N = 5; 2*AN1, 2*AN2, 1*AN3)
**0–1000 Hz/200–750 Hz**	**0.31** (N = 7; 7*TN1)	
**0–1000 Hz/0–100 Hz**	**0.953** (N = 9; 4*BSN1, 5*TN1)	**0.124** (N = 13; 2*AN1, 3*AN2, 5*AN3, 1* AN7, 2*AN12)
**0–1000 Hz/100–200 Hz**	**0.176** (N = 7; 2*BSN1, 5*TN1)	**0.893** (N = 5; 1*AN1, 1*AN2, 2*AN3, 1*AN12)
**0–100 Hz/100–200 Hz**	**0.021** (N = 9; 3*BSN1, 1*USN1, 5*TN1)	**0.463** (N = 6; 1*AN1, 1*AN2, 3*AN3; 1*AN12)
**100–500 Hz/200–750 Hz**	**0.028** (N = 7; 7*TN1)	(**N = 1**; 1*AN3)

Due to multiple testing the significance threshold was set at p = 0.007 (according to Bonferroni).

In summary, a degradation of the song envelope with very different frequency bands influenced the neuronal representation of an amplitude modulated stimulus in a similar way. This is in accordance with most behavioural results (Fig. 2), with the potential exception of the 0–100 Hz band (Figure 3).

## Discussion

A basic problem for acoustically communicating animals is noise on different time scales [1], [4], [7], [8], [34], [35]. Correspondingly, we find various mechanisms implemented, both on the sender's and on the receiver's side, which aim to improve signal transmission and signal detection [4], [7], [10], [11], [35]. Sharply tuned auditory filters may improve signal detection by reducing the amount of ambient high frequency noise relative to the signal of interest [36], [37]. However, in many species signal recognition depends on the pattern of amplitude modulations, and may be affected by noise in the low frequency range. Hence, the question is whether neuronal filter mechanisms exist that are tuned to the frequency range of amplitude modulations. In the auditory pathway of mammals indeed a bank of neuronal filters has been described that are tuned to different modulation frequencies [38], [39], [40]. Whether a similar type of filtering could help also in insects to reduce masking energy in the frequency domain of amplitude modulations, however, is not clear.

Some species of the acridid subfamily Gomphocerinae have a bidirectional communication system. Females inclined to mate respond to the males' calling song, whereby the male himself is able to approach the female by phonotaxis. For *Chorthippus biguttulus* the recognition of species and sex relies predominantly on the modulation patters of their songs [17], [26], [41]. The rather broad frequency tuning of their auditory receptors (best frequencies around 5–7 Hz or 15–25 Hz) merely enables them to recognize sex specific differences in the carrier frequencies of the songs [26], [42]. However, an analysis of subtle differences in carrier spectra is highly unlikely [43]. Hence the communication system of *C. biguttulus* provides an excellent model to investigate noise tolerance conveyed by mechanisms operating in the temporal domain [15], [17], [18].

There are two major factors that lead to a degradation of the temporal pattern of songs, both with an emphasis on low modulations frequencies: (i) reflections and reverberations during the sound propagation in the vegetation (see [2], [44]), and (ii) songs of conspecifics or of other species with similar AM spectra (cf. [37], [45], [46]). *C. biguttulus* lives in sometimes dense aggregations of hundreds of animals [47], often sympatric with other species, and neither males nor females synchronize their songs. As a consequence, the most severe masking problems will be caused by conspecific signallers because not only the spectra of the masking sounds and the signal overlap but also the sound pulses produced by nearby singing males lead to a degradation of the AM pattern (see [2]). But also the songs of different species share similar AM spectra, mostly below 100 Hz and thus may degrade the song pattern [22], [45].

To test both – the impact on signal recognition of behaving animals and the impact on underlying auditory processing mechanisms - a female song was used, whose temporal pattern was disturbed by random amplitude modulations. By applying envelope maskers with various band widths we investigated whether distinct modulation frequencies have more pronounced adverse effects on signal recognition than others. This approach allowed us to test whether auditory neurones of grasshoppers that respond selectively to certain amplitude modulations [19], [20], [21], may contribute to improve the recognition of degraded signals.

For most males the critical degradation level, at which song recognition failed, was found between −6 and 0 dB (Figure 2B, C). Different noise bands showed no significant differences in their impact on song recognition, with one exception: the 0–100 Hz envelope masker. This masker was expected to have the strongest detrimental impact on signal recognition due to its complete overlap with the signal's AM frequencies and the response ranges of the auditory neurones. However, most animals continued to respond to the masked songs, no matter how strong the degradation was. The animals seem to have classified fragments of these random amplitude modulations as resembling female songs although the modulations obviously did not coincide with the original song structure. Since the main envelope frequencies for the specific female song were between 0–100 Hz (Figure 1B), the most likely explanation for this behaviour is that the random amplitude modulations within this frequency band by chance resulted in envelope structures that activated the male's song recognition network. Two additional observations make this interpretation plausible: (i) earlier studies have shown that *Chorthippus biguttulus* males respond already to very short segments of a female song, a 165 to 250 ms segment being sufficient for signal recognition [27], [33], and (ii) males are more tolerant than females to deviations from the normal syllable-to-pause pattern (see Fig. 11 in [26]). This assumption was further supported by the control experiment in which males showed strong phonotactic responses to a stimulus consisting of pure random amplitude modulations (0–75 Hz), without any song (column **n** in Figure 3B).

The second part of our study aimed at finding neuronal correlates that can explain the similar impairment of signal recognition caused by different envelope maskers. We performed intracellular recordings on auditory receptors, local interneurons, and ascending neurons to test whether envelope noise with different frequency ranges would differently impede the neuronal representation of acoustic stimuli. We hypothesized that the impact an envelope masker has on the neuronal representation would depend on the temporal filter characteristics of the neuron investigated. Neuronal filter properties are commonly described by modulation transfer functions (MTFs) [38]. The rate modulation transfer function (rMTF) characterizes how a neuron's firing rate changes at various modulation frequencies. Experiments with sinusoidal amplitude modulated (SAM) stimuli in locusts have shown that receptors and the majority of local neurons exhibit an all-pass or at least broad low pass rMTF characteristic, while many of the ascending neurons show low-pass or band-stop properties [19], [20], [21]. If the frequency components of an envelope masker came to lie beyond filter range of a neuron, one could expect that these modulations have a minor impact on the neural representation of the original signal. The corner frequencies of the majority of the ascending neurones were found to lie below 100 Hz [20], [21]. Hence, for the ascending neurons we hypothesized that the narrow band envelope masker (0–100 Hz) would have the strongest impact on spike trains, followed by the two broadband maskers (0–1000 Hz and 0–1000 Hz notch) that have a partial overlap to the signal's modulation frequency range. The other maskers (100–200 Hz; 100–500 Hz and, in particular, 200–750 Hz) were expected to have less detrimental effects. Because the AM filters of local neurons are, as a rule, much broader, we expected that the detrimental effects of different noise bands would differ less for local neurons. However, the analysis of spike train distances by the van Rossum metric [31] revealed only minor differences between different envelope maskers for both, ANs and LNs (Figure 5). These neurophysiological data thus are in line with the majority of behavioural results, which also yielded no significant differences between most envelope maskers.

The 0–100 Hz envelope masker was an obvious exception. The males responded vigorously also to severely degraded signals (Figure 3) although the corresponding spike train distances for this envelope masker indicate a similar degradation level of the underlying neuronal representation as for other frequency bands (Figure 4B,F; Figure 5D,F). This discrepancy between the behavioural and the neurophysiological results highlights once more the complexity of the pattern recognition system of this grasshopper species [17], [18], [48]. On the other hand, it also reveals a potential limitation of the spike train metric approach. Earlier behavioural experiments have demonstrated that *C. biguttulus* does accept model songs in which the rhythm of syllables and pauses was manipulated, provided that a sufficient amount of short segments with the correct syllable-to-pause pattern is present [17]. Obviously, signal recognition does not depend on a simple cross correlation with a stored template [17]. Rather these results suggest that within the space of potential stimuli there exists an extended region of attractive stimuli [17], [48]. As mentioned above, the males may have responded to the pure noise stimulus or to a female song that was severely degraded with the 0–100 Hz band (see Figure 3) because these stimuli by chance contained short segments of amplitude modulations that belonged to an attractive region in the stimulus space. If this is true, then the method of computing metric distances between spike trains – taken over the total length of the spike trains – may overlook the critical stimulus segments relevant for recognition.

The comparison of behavioural and neurophysiological results presented here indicates that *C. biguttulus* is not able to eliminate perturbing envelope noise by means of neuronal filters that are tuned to certain amplitude modulation frequencies. Since peripheral filter mechanisms working in the range of carrier frequencies can also play only a minor role in acridid grasshoppers [15], the animals have to rely on behavioural strategies to cope with signal degradation occurring in their natural habitat [10].

## Supporting Information

Figure S1 **Amplitude spectrum of the envelope of the original female song degraded at 0 dB with different frequency bands of envelope noise.** A) 0–1000 Hz B) 200–750 Hz C) 100–200 Hz D) 0–100 Hz. Although the four graphs show the same degradation level of 0 dB, meaning that the original signal was degraded with the same amount of noise energy, the disturbance of the fourier components of the original signals is quite different (compare with Figure 1C). A neuronal filter rejecting amplitude modulations beyond 100 Hz, could substantially decrease the noise components for A, B, C but not for D.
(DOC)

## References

[1] Richards DG, Wiley RH (1980). Reverberations and amplitude fluctuations in the propagation of sound in a forest: implications for animal communication.. The American Naturalist.

[2] Lang F (2000). Acoustic communication distances of a gomphocerine grasshopper.. Bioacoustics.

[3] Römer H, Barth F, Schmid A (2001). Ecological constraints for sound communication: From grasshoppers to elephants.. Ecology of sensing.

[4] Brumm H, Slabbekoorn H (2005). Acoustic communication in noise.. Advanced Studies of Behaviour.

[5] Römer H, Lewald J (1992). High-frequency sound transmission in natural habitats: Implications for the evolution of insect acoustic communication.. Behavioral Ecology and Sociobiology.

[6] Einhäupl A, Stange N, Hennig RM, Ronacher B (2011). Attractiveness of grasshopper songs correlates with their robustness against noise.. Behavioral Ecology.

[7] Wiley RH (2006). Signal detection and animal communication.. Advanced Studies of Behaviour.

[8] Bradbury JW, Vehrencamp SL (1998).

[9] Ronacher B, Hoffmann C (2003). Influence of amplitude modulated noise on the recognition of communication signals in the grasshopper *Chorthippus biguttulus.*. Journal of Comparative Physiology A: Neuroethology, Sensory, Neural, and Behavioral Physiology.

[10] Römer H, Bailey W, Dadour I (1989). Insect hearing in the field. III Masking by noise.. Journal of Comparative Physiology A: Neuroethology, Sensory, Neural, and Behavioral Physiology.

[11] Bee MA (2008). Finding a mate at a cocktail party: spatial release from masking improves acoustic mate recognition in grey treefrogs.. Animal Behaviour.

[12] Bee MA, Schwartz JJ (2009). Behavioral measures of signal recognition thresholds in frogs in the presence and absence of chorus-shaped noise.. Journal of the Acoustical Society of America.

[13] Bregman AS (1990). Auditory scene analysis: The perceptual organization of sound.

[14] Klump GM, Kroodsma DE, Miller EH (1996). Bird communication in the noisy world.. Ecology and Evolution of Acoustic Communication in Birds.

[15] Hennig RM, Franz A, Stumpner A (2004). Processing of auditory information in insects.. Microscopy Research and Technique.

[16] Machens CK, Stemmler MB, Prinz P, Krahe R, Ronacher B (2001). Representation of Acoustic Communication Signals by Insect Auditory Receptor Neurons.. The Journal of Neuroscience.

[17] von Helversen D, von Helversen O (1998). Acoustic pattern recognition in a grasshopper: processing in the time or frequency domain?. Biological Cybernetics.

[18] Schmidt AKD, Ronacher B, Hennig R (2008). The role of frequency, phase and time for processing of amplitude modulated signals by grasshoppers.. Journal of Comparative Physiology A: Neuroethology, Sensory, Neural, and Behavioral Physiology.

[19] Wohlgemuth S, Ronacher B (2007). Auditory discrimination of amplitude modulations based on metric distances of spike trains.. Journal of Neurophysiology.

[20] Weschke G, Ronacher B (2008). Influence of sound pressure level on the processing of amplitude modulations by auditory neurons of the locust.. Journal of Comparative Physiology A: Neuroethology, Sensory, Neural, and Behavioral Physiology.

[21] Wohlgemuth S, Vogel A, Ronacher B (2011). Encoding of amplitude modulations by auditory neurons of the locust: influence of modulation frequency, rise time, and modulation depth.. Journal of Comparative Physiology A: Neuroethology, Sensory, Neural, and Behavioral Physiology.

[22] Clemens J, Weschke G, Vogel A, Ronacher B (2010). Intensity invariance properties of auditory neurons compared to the statistics of relevant natural signals in grasshoppers.. Journal of Comparative Physiology A: Neuroethology, Sensory, Neural, and Behavioral Physiology.

[23] Ronacher B, Stumpner A (1988). Filtering of behaviourally relevant temporal parameters of a grasshopper's song by an auditory interneuron.. Journal of Comparative Physiology A: Neuroethology, Sensory, Neural, and Behavioral Physiology.

[24] Neuhofer D, Wohlgemuth S, Stumpner A, Ronacher B (2008). Evolutionarily conserved coding properties of auditory neurons across grasshopper species.. Proceedings of the Royal Society B: Biological Sciences.

[25] Neuhofer D, Stemmler M, Ronacher B (2011). Neuronal precision and the limits for acoustic signal recognition in a small neuronal network.. Journal of Comparative Physiology A: Neuroethology, Sensory, Neural, and Behavioral Physiology.

[26] von Helversen D, von Helversen O (1997). Recognition of sex in the acoustic communication of the grasshopper *Chorthippus biguttulus* (Orthoptera, Acrididae).. Journal of Comparative Physiology A: Sensory, Neural, and Behavioral Physiology.

[27] Ronacher B, Krahe R, Hennig RM (2000). Effects of signal duration on the recognition of masked communication signals by the grasshopper *Chorthippus biguttulus.*. Journal of Comparative Physiology A: Neuroethology, Sensory, Neural, and Behavioral Physiology.

[28] Vogel A, Ronacher B (2007). Neural Correlations Increase Between Consecutive Processing Levels in the Auditory System of Locusts.. Journal of Neurophysiology.

[29] Römer H, Marquart V (1984). Morphology and physiology of auditory interneurons in the metathoracic ganglion of the locust.. Journal of Comparative Physiology A: Neuroethology, Sensory, Neural, and Behavioral Physiology.

[30] Stumpner A, Ronacher B (1991). Auditory Interneurones in the Metathoracic Ganglion of the Grasshopper *Chorthippus biguttulus*: I. Morphological and Physiological Characterization.. The Journal of Experimental Biology.

[31] van Rossum MCW (2001). A Novel Spike Distance.. Neural Computation.

[32] Machens CK, Schuetze H, Franz A, Kolesnikova O, Stemmler MB (2003). Single auditory neurons rapidly discriminate conspecific communication signals.. Nature Neuroscience.

[33] Ronacher B, Krahe R (1998). Song recognition in the grasshopper *Chorthippus biguttulus* is not impaired by shortening song signals: implications for neuronal encoding.. Journal of Comparative Physiology A: Sensory, Neural, and Behavioral Physiology.

[34] Buus S, MJ C (1998). Auditory Masking.. Handbook of acoustics.

[35] Klump GM, Baur A (1990). Intensity discrimination in the European starling (*Sturnus vulgaris*).. Naturwissenschaften.

[36] Kostarakos K, Hennig RM, Römer H (2009). Two matched filters and the evolution of mating signals in four species of cricket.. Frontiers in Zoology.

[37] Schmidt AKD, Römer H (2011). Solutions to the Cocktail Party Problem in Insects: Selective Filters, Spatial Release from Masking and Gain Control in Tropical Crickets.. PLoS ONE.

[38] Joris PX, Schreiner CE, Rees A (2004). Neural Processing of Amplitude-Modulated Sounds.. Physiological Reviews.

[39] Langner G (1992). Periodicity coding in the auditory system.. Hearing Research.

[40] Langner G, Schreiner CE (1988). Periodicity coding in the inferior colliculus of the cat. I. Neuronal mechanisms.. Journal of Neurophysiology.

[41] von Helversen D, von Helversen O (1994). Forces driving coevolution of song and song recognition in grasshoppers.. Fortschritte der Zoologie.

[42] Römer H (1976). Die Informationsverarbeitung tympanaler Rezeptorelemente von *Locusta migratoria*.. Journal of Comparative Physiology A: Neuroethology, Sensory, Neural, and Behavioral Physiology.

[43] Stumpner A, von Helversen D (2001). Evolution and function of auditory systems in insects,.. Naturwissenschaften.

[44] Michelsen A, Larsen ON, Huber F, Markl H (1983). Strategies for acoustic communication in complex environments.. Neuroethology and behavioral physiology.

[45] Römer H, Bailey W (1998). Strategies for hearing in noise: peripheral control over auditory sensitivity in the bushcricket *Sciarasaga quadrata* (Austrosaginae: Tettigoniidae).. Journal of Experimental Biology.

[46] Römer H, Krusch M (2000). A gain-control mechanism for processing of chorus sounds in the afferent auditory pathway of the bushcricket *Tettigonia viridissima* (Orthoptera; Tettigoniidae).. Journal of Comparative Physiology A: Neuroethology, Sensory, Neural, and Behavioral Physiology.

[47] Kriegbaum H (1989). Female choice in the grasshopper *Chorthippus biguttulus*.. Naturwissenschaften.

[48] Balakrishnan R, von Helversen D, von Helversen O (2001). Song pattern recognition in the grasshopper *Chorthippus biguttulus*: the mechanism of syllable onset and offset detection.. Journal of Comparative Physiology A: Sensory, Neural, and Behavioral Physiology.

