# Supplementary material for: Influence of Different Envelope Maskers on Signal Recognition and Neuronal Representation in the Auditory System of a Grasshopper
Source: PLoS One. 2012 Mar 30;7(3):e34384. doi: 10.1371/journal.pone.0034384 (PMC3316687; doi:10.1371/journal.pone.0034384)
Supplement: Figure S1 — Amplitude spectrum of the envelope of the original female song degraded at 0 dB with different frequency bands of envelope noise. A) 0–1000 Hz B) 200–750 Hz C) 100–200 Hz D) 0–100 Hz. Although the four graphs show the same degradation level of 0 dB, meaning that the original signal was degraded with the same amount of noise energy, the disturbance of the fourier components of the original signals is quite different (compare with Figure 1C). A neuronal filter rejecting amplitude modulations beyond 100 Hz, could substantially decrease the noise components for A, B, C but not for D. (DOC) [file pone.0034384.s001.doc]

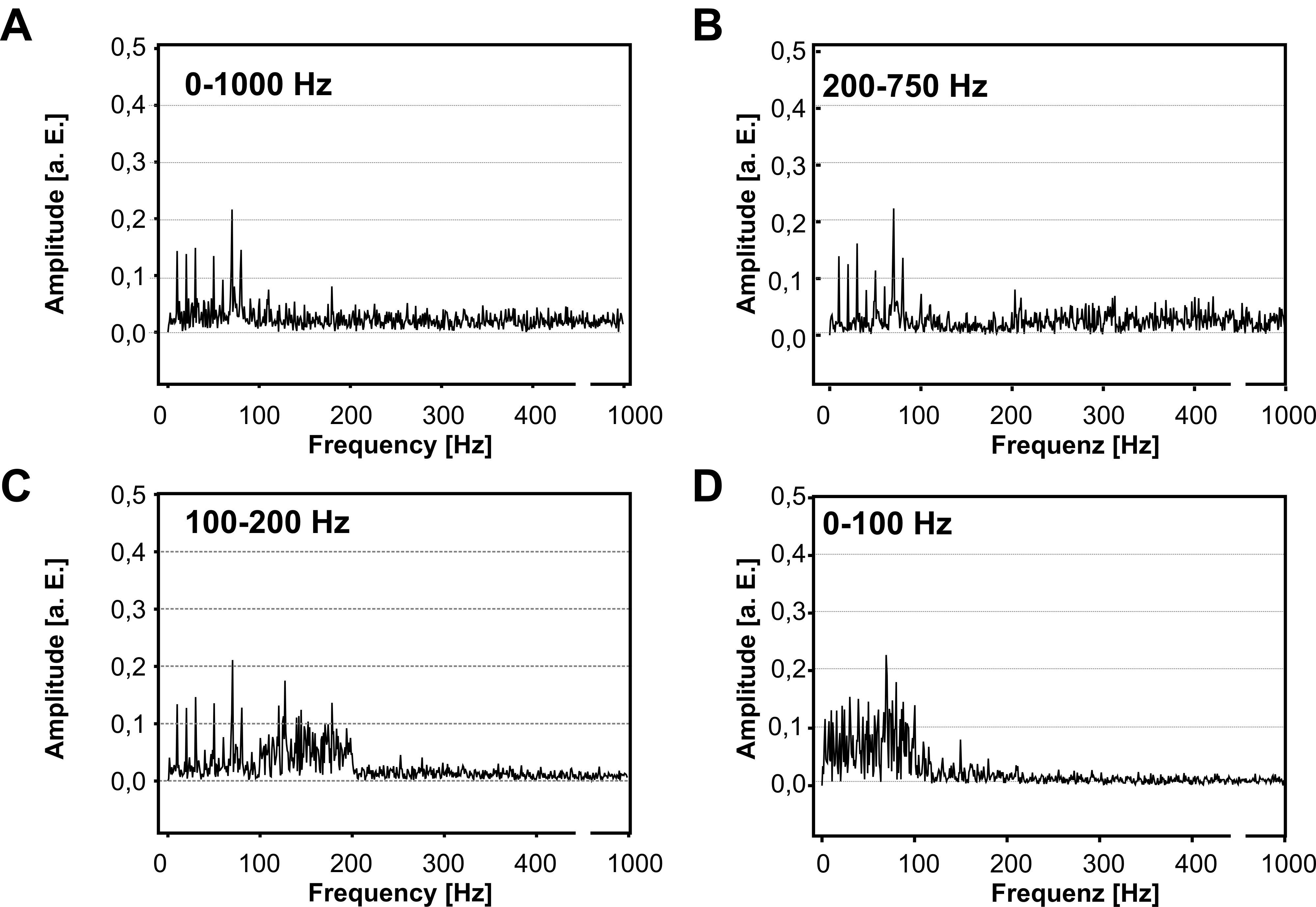


**Figure S1. Amplitude spectrum of the envelope of the original female song degraded at 0 dB with different frequency bands of envelope noise.** A) 0-1000 Hz B) 200-750 Hz C) 100-200 Hz D) 0-100 Hz. Although the four graphs show the same degradation level of 0 dB, meaning that the original signal was degraded with the same amount of noise energy, the disturbance of the fourier components of the original signals is quite different (compare with Figure 1C). A neuronal filter rejecting amplitude modulations beyond 100 Hz, could substantially decrease the noise components for A, B, C but not for D.
